# Delayed Mismatch Field Latencies in Autism Spectrum Disorder with Abnormal Auditory Sensitivity: A Magnetoencephalographic Study

**DOI:** 10.3389/fnhum.2017.00446

**Published:** 2017-09-06

**Authors:** Junko Matsuzaki, Kuriko Kagitani-Shimono, Hisato Sugata, Ryuzo Hanaie, Fumiyo Nagatani, Tomoka Yamamoto, Masaya Tachibana, Koji Tominaga, Masayuki Hirata, Ikuko Mohri, Masako Taniike

**Affiliations:** ^1^Molecular Research Center for Children's Mental Development, United Graduate School of Child Development, Osaka University Graduate School of Medicine Osaka, Japan; ^2^Division of Developmental Neuroscience, United Graduate School of Child Development, Osaka University Graduate School of Medicine Osaka, Japan; ^3^Department of Pediatrics, Osaka University Graduate School of Medicine Osaka, Japan; ^4^Department of Neurosurgery, Osaka University Graduate School of Medicine Osaka, Japan; ^5^Endowed Research Department of Clinical Neuroengineering, Global Center for Medical Engineering and Informatics, Osaka University Osaka, Japan

**Keywords:** autism spectrum disorders (ASD), abnormal auditory sensitivity, mismatch fields, magnetoencephalography (MEG), oddball paradigm

## Abstract

Although abnormal auditory sensitivity is the most common sensory impairment associated with autism spectrum disorder (ASD), the neurophysiological mechanisms remain unknown. In previous studies, we reported that this abnormal sensitivity in patients with ASD is associated with delayed and prolonged responses in the auditory cortex. In the present study, we investigated alterations in residual M100 and MMFs in children with ASD who experience abnormal auditory sensitivity. We used magnetoencephalography (MEG) to measure MMF elicited by an auditory oddball paradigm (standard tones: 300 Hz, deviant tones: 700 Hz) in 20 boys with ASD (11 with abnormal auditory sensitivity: mean age, 9.62 ± 1.82 years, 9 without: mean age, 9.07 ± 1.31 years) and 13 typically developing boys (mean age, 9.45 ± 1.51 years). We found that temporal and frontal residual M100/MMF latencies were significantly longer only in children with ASD who have abnormal auditory sensitivity. In addition, prolonged residual M100/MMF latencies were correlated with the severity of abnormal auditory sensitivity in temporal and frontal areas of both hemispheres. Therefore, our findings suggest that children with ASD and abnormal auditory sensitivity may have atypical neural networks in the primary auditory area, as well as in brain areas associated with attention switching and inhibitory control processing. This is the first report of an MEG study demonstrating altered MMFs to an auditory oddball paradigm in patients with ASD and abnormal auditory sensitivity. These findings contribute to knowledge of the mechanisms for abnormal auditory sensitivity in ASD, and may therefore facilitate development of novel clinical interventions.

## Introduction

Autism spectrum disorder (ASD) is a neurodevelopmental disorder characterized primarily by impaired social and communication skills, and by repetitive and stereotyped behavior (American Psychiatric Association, 2000). In addition to these classical features, the Diagnostic and Statistical Manual of Mental Disorders, 5th edition (DSM-5) describes sensory abnormalities (i.e., hyper and/or hypo reactivity to sensory input) as novel diagnostic features of ASD (American Psychiatric Association, 2013). ASD is considered as a heterogeneous group of neurodevelopmental disorders and, therefore, it is important to focus on ASD subtypes to elucidate the underlying neural bases of their defining characteristics.

Abnormal auditory sensitivity is the most common sensory impairment in ASD. However, individuals with abnormal auditory sensitivity simultaneously show both over- (e.g., fear, crying, covering ears with hands) and under-response (e.g., appears not to hear, unresponsive) to environmental sounds (Dunn, 2002; Lane et al., 2010, 2011). These abnormalities interrupt behavioral adaptation (e.g., refusal to come into a classroom or supermarket) and may contribute to behavioral problems in children with ASD (Lane et al., 2010, 2011). However, the neural bases of these sensory abnormalities remain unclear.

Previous electrophysiological studies have reported altered cortical responses to tone stimuli in the auditory cortex of children with ASD (6–14 years old) compared with typically developing (TD) children (Gage et al., 2003; Edgar et al., 2014, 2015). In contrast, there has been no previous research to elucidate differential cortical activation depending on the presence of abnormal auditory sensitivity (i.e., differences between children who show abnormal auditory sensitivity and children who do not show such abnormalities). Furthermore, we first demonstrated with magnetoencephalography (MEG) an association of abnormal auditory sensitivity with delayed peak M50/M100 latencies (Matsuzaki et al., 2012), increased M50 dipole moments over time, and prolongation of response durations elicited by repeated auditory stimuli. From such results, we concluded that maturational abnormalities and abnormal thalamic sensory gating were distinctive features of children with ASD who have abnormal auditory sensitivity (Matsuzaki et al., 2014).

Furthermore, we have also highlighted that such sensory phenomena might be related to neural abnormalities not only in the primary auditory area but also in other temporal and frontal areas. Frontal areas are crucial for attentional processing and inhibitory control processing (Aron et al., 2014; Stramaccia et al., 2015). Auditory mismatch negativity (MMN) or mismatch fields (MMFs), measured with electroencephalography (EEG), or MEG, reflect neural discrimination responses in the acoustic environment outside the focus of attention (Alho, 1995; Naatanen et al., 2014). They are calculated by subtracting responses to standard stimuli (frequent sounds) from those to deviant stimuli (infrequent sounds, occurring occasionally among frequently repeated sounds; Pulvemuller and Shtyrov, 2006), and are mainly located in temporal and frontal areas (Mamashli et al., 2017).

Several electrophysiological studies have reported on MMN or MMFs in children with ASD. For example, Gomot et al. (2002) observed earlier MMN peak latencies to deviant stimuli using tones in children with ASD (5–9 years old) compared with TD children. In addition, Seri et al. (1999) observed significantly smaller MMN responses and longer latencies to tones in children with tuberous sclerosis complex associated with autistic behavior (7–10 years old) compared with TD children. Furthermore, Dunn et al. (2008) reported that MMN to tones in children with ASD (6–12 years old) were reduced compared with TD children. In addition, an absence of MMF to speech and non-speech stimuli was observed in 4–6 year-old children with ASD compared with matched controls (Galilee et al., 2017). However, results regarding MMN/MMFs in participants with ASD are inconsistent, probably due to the heterogeneity of ASD symptoms, including the severity of sensory abnormalities.

In the present study, we aimed to clarify the relationships between MMF cortical activations in both temporal and frontal areas and the severity of abnormal auditory sensitivity, with MEG in children with ASD. We hypothesized that abnormal discrimination processing of sounds and abnormal attention switching would be associated with abnormal auditory sensitivity in patients with ASD.

## Methods

### Participants

Twenty boys diagnosed with ASD and 13 age-matched TD boys participated in this study (Table 1). Clinical participants were recruited from Osaka University Hospital or other hospitals in Osaka Prefecture. TD children were recruited through a public newsletter distributed throughout Osaka prefecture. Children who had received special education services or had a history of neurological disorders in conjunction with a diagnosis of developmental disorders were excluded from the TD group. We also confirmed a lack of autistic traits in this group using the Japanese version of the Autism Screening Questionnaire (ASQ; 21, 22). All participants had normal hearing confirmed by medical records or parental report.

**Table 1:** Characteristics of study participants.

**Group**	**TD children (*n* = 13)**	**ASD without abnormal auditory sensitivity (*n* = 9)**	**ASD with abnormal auditory sensitivity (*n* = 11)**
	**Mean ± SD**	**Mean ± SD**	**Mean ± SD**
Age (years)	9.46 ± 1.51	9.07 ± 1.31	9.62 ± 1.82
FIQ	–	101.11 ± 13.00	105.36 ± 15.89
ASQ	2.08 ± 1.56^*^^#^	14.38 ± 4.47^*^	15.00 ± 5.37^#^
SP auditory item scores	38.69 ± 1.75^*^	32.33 ± 2.78^#^	23.00 ± 5.00^*^^#^
CBCL attention scores^†^	–	65.75 ± 3.81	71.36 ± 10.87

*TD, typically developing; ASD, autism spectrum disorder; SD, standard deviation; FIQ, full-scale intelligence quotient of Wechsler Intelligence Scale for Children, 3rd version; ASQ, Autism Screening Questionnaire (cut-off ≧ 13)*.

* p < 0.01 for comparison between TD and ASD without abnormal auditory sensitivity groups,

# *p < 0.01 for comparison between TD and ASD with abnormal auditory sensitivity groups. SP, Sensory Profile (cut-off ≥ 30)*.

* p < 0.01 for comparison between TD and ASD with abnormal auditory sensitivity groups,

# p < 0.01 for comparison between ASD without and with abnormal auditory sensitivity groups. CBCL, Child Behavior Checklist (

† *ASD without abnormal auditory sensitivity group, n = 8; ASD with abnormal auditory sensitivity group, n = 11)*.

ASD was diagnosed by experienced clinicians according to DSM-5 criteria (American Psychiatric Association, 2013), and diagnoses were confirmed with the Autism Diagnostic Observation Schedule-Generic (ADOS-G; Lord et al., 2000). Children were assessed for autism using the ASQ (Berument et al., 1999; Dairoku et al., 2004). The Wechsler Intelligence Scale for Children, 3rd edition (WISC-III) was also administered. Abnormal auditory sensitivity was assessed in all children with the Japanese version of the Sensory Profile (SP; 5). Participants with ASD were categorized into two groups based on auditory item scores (cut-off ≥ 30). ASD with abnormal auditory sensitivity was categorized as *SP* < 30, while ASD without abnormal auditory sensitivity was categorized as *SP* ≥ 30. The attention items of the Japanese version of the Child Behavior Checklist (CBCL) were used to assess attention in participants with ASD (Achenbach, 1991; Itani et al., 2001). The ASQ, SP, and CBCL are standardized caregiver questionnaires. All participants had no medication on the day of behavioral testing, and MEG and MRI measurements.

All participants and guardians provided written informed consent to participate in accordance with the principles of the Declaration of Helsinki. This study was conducted in accordance with the guidelines of the Institutional Review Board of Osaka University Hospital, Osaka University, Japan, who approved the study protocol. Participants received a gift card as compensation for participation.

### Auditory stimuli

The auditory oddball stimuli and tone pip stimuli were calibrated at 75–80 dB, and binaurally presented to participants via a sound pressure transducer and sound conduction tubing leading to the auditory canal via ear tip inserts. For MMF responses, standard and deviant stimuli were 300 ms sinusoidal tones with frequencies of 300 Hz (probability = 83.5%) and 700 Hz (probability = 16.5%), respectively, which were emitted by a presentation system (Presentation, Neurobehavioral System, Inc. San Francisco, CA, USA). A total of 167 standard and 33 deviant stimuli were presented, with an inter-stimulus interval of 2,500–3,000 ms.

### MEG and magnetic resonance imaging (MRI) measurements

Prior to MEG recordings, we scanned the 3 dimensional (3D) facial surface of each participant (Fast SCAN CobraTM, POLHEMUS, ARANTZ Scanning Limited, Christchurch, New Zealand) with 5 head-marker coils as fiduciary points (the external meatus of each ear, two points on the forehead, and the nasion). While lying down on a bed in a magnetically shielded room, participants were examined by a 160-channel whole-head MEG system equipped with SQUID gradiometers (PQ 1160C, Yokogawa Electric Corporation, Tokyo, Japan). The positions of the head-marker coils were obtained before and after each recording to evaluate head movement (Sugata et al., 2012). Data were acquired at a sampling rate of 1,000 Hz. Before the MEG recording, we provided the following instruction to children: “Please relax and do not move your head or body. Look at the monitor, if you feel uncomfortable, please raise your hands up.” We then monitored the children during the experiment with a video camera. We used a non-attentive listening condition with a visual fixation point.

Individual anatomical MRI data were obtained with a 3.0 Tesla whole-body magnetic resonance scanner equipped with a standard whole-head coil (Signa Excite HD, GE Healthcare, Milwaukee, USA). A 3D T1-weighted axial protocol was used, with imaging parameters as follows: 3D-spoiled GRASS sequence, repetition time (TR) = 10.1 ms; echo time (TE) = 3.0 ms; flip angle = 18°; field of view (FOV) = 220 × 220 mm^2^; matrix size = 320 × 256; slice thickness = 1.4 mm; and number of excitations (NEX) = 1.A 3D T1 (Hanaie et al., 2013). Using superimposition and registration of 3D facial surface data and fiduciary points on individual MRI images, MEG data were superimposed on individual MRIs with an anatomical accuracy of 2–3 mm.

To determine the activity of each brain region, including the temporal and frontal areas, we used Brainstorm software, which is freely available for download online under the GNU general public license (http://neuroimage.usc.edu/brainstorm; Tadel et al., 2011). Each individual MRI was reconstructed using Freesurfer 5.3.0 image analysis software (http://surfer.nmr.mgh.harvard.edu/; Fischl, 2012). To remove the 60 Hz frequency and harmonics from the continuous files, we applied notch filters. Epochs with artifacts > 2,000 fT/cm were excluded. Furthermore, artifacts caused by heartbeats and eye movements were excluded using signal space projections (Tadel et al., 2011). After reducing noise, the remaining data were arithmetically averaged and z-score normalization was applied.

**Figure 1:**
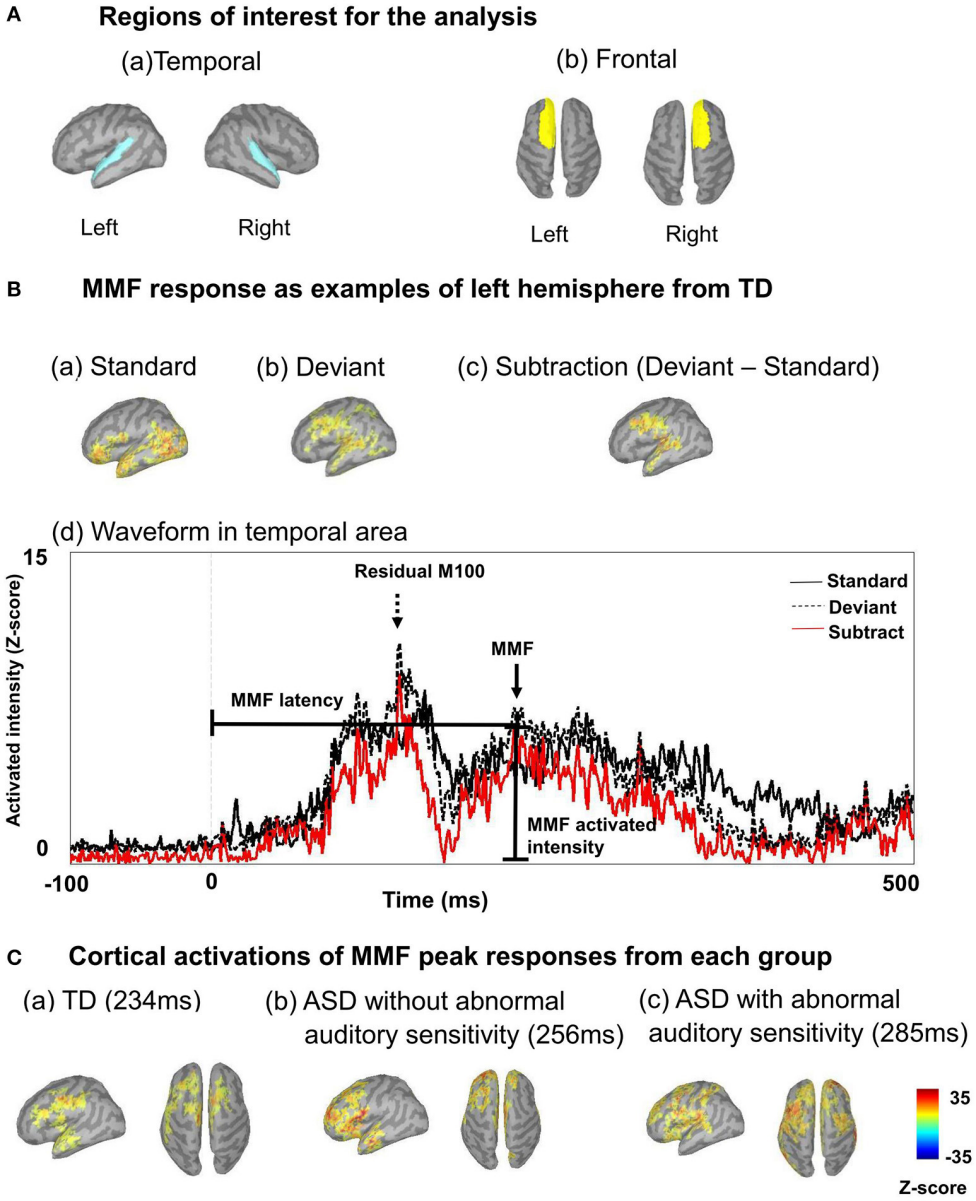
**(A)** Regions of interest are shown, including left temporal and right temporal **(a)**, and left frontal and right frontal regions **(b)**. **(B)** Mismatch fields (MMF) of example responses are shown from the left hemisphere of a typically developing participant, including activated intensity in standard conditions **(a)**, and activated intensity in deviant conditions **(b)**. To determine MMFs, we subtracted activated intensities in standard conditions from those in deviant conditions **(c)**. Example waveforms are shown from the left hemisphere of a typically developing participant **(d)**. Arrows indicate MMFs, dashed arrows indicate the residual M100. Lines indicate MMF latency and activated intensity. Vertical lines on the averaged waveform trace indicate stimulus onset (0 ms). The black line indicates cortical activation of the standard condition, the dashed black line indicates deviant and the red line indicates subtracted cortical activation. **(C)** Example cortical activations of MMF peak responses from each group are shown (**a**: TD 234 ms; **b**: ASD without abnormal auditory sensitivity 256 ms; **c**: ASD with abnormal auditory sensitivity 285 ms). Delayed MMF responses can be observed in ASD with abnormal auditory sensitivity.

We performed source estimation with weighted minimum-norm estimation (wMNE), which used an algorithm adapted from depth-weighted minimum linear L2 norm estimators conducted with MNE software (Hämäläinen, 2009; Tadel et al., 2011). Subsequently, data were grand averaged individually and images were projected onto the Colin 27 average brain template (an MNI brain with 1 mm resolution) for standard and deviant conditions. We used an overlapping-sphere model (Mosher et al., 1999; Tadel et al., 2011). A total of 162–167 standard and 28–33 deviant MMFs were used for grand averaged data, individually. The temporal and frontal areas were used to determine regions of interest for the analyses, based on the Desikan-Killiany atlas (Figure 1A). Regions of interest and examples from a TD participant are shown in Figure 1. To determine the residual M100/MMF latencies and activated intensities, which is a similar index to amplitudes (Doucet et al., 2012; Strauss et al., 2015), we subtracted the values of the standard condition from those of the deviant condition (Figure 1B). We display the time series of cortical activations from each ROI individually for standard deviant and subtracted data (Figures 1Bd). These data were exported as an ASCII file for further analysis. The timeframes of analysis of cortical activation were defined from 100 ms before stimulation to 350 ms after stimulation. We identified the first maximum peak as the residual M100 peaks and the last as the MMF peak observed 350 ms after stimulus onset (Figures 1B,C). Finally, data were grand averaged by group. Analysis of variance (ANOVA) was used to assess the effects of hemispheres and groups on full-scale IQ (FIQ), SP, attention scores, residual M100/MMF latencies, and activated intensities. Finally, Bonferroni's correction was applied to *post-hoc* analyses. To determine associations between residual M100/MMF latencies and severity of sensory and attentional abnormalities, SP and attention scores were used in the correlation analysis. Partial correlations were performed covaried for age. All analyses were performed with SPSS version 22.0 (IBM, Tokyo, Japan), and the level of significance was set at *p* < 0.05.

## Results

### Demographics

There was no main effect of age [*F*_(2, 30)_ = 0.32, *p* = 0.731; Table 1]. However, there was a significant main effect of group on ASQ scores [*F*_(2, 28)_ = 31.99, *p* < 0.001, η^2^ = 0.70], and the ASQ scores of the ASD groups with and without abnormal auditory sensitivity were significantly higher than those of the TD group (*p* < 0.001). ASQ scores did not differ between the two ASD groups. The auditory score for SP revealed a significant main effect of group [*F*_(2, 30)_ = 63.30, *p* < 0.001, η^2^ = 0.81]. As predicted, individuals with ASD with abnormal auditory sensitivity had lower SP scores, indicating more severe abnormal auditory sensitivity compared with the ASD group without auditory sensitivity or the TD group (*p* < 0.001). WISC-III scores did not differ between the ASD groups with or without abnormal auditory sensitivity [*t* = −0.650, *p* = 0.530]. Individuals with ASD with abnormal auditory sensitivity had higher attention scores compared with the ASD without auditory sensitivity group (mean ± standard deviation = 65.75 ± 3.81 and 71.36 ± 10.87, respectively). However, this difference was not significant [*t* = −1.390, *p* = 0.102]. SP scores were not significantly correlated with attention scores [*r* = −0.383, *p* = 0.105].

### Residual M100 latencies

**Figure 2:**
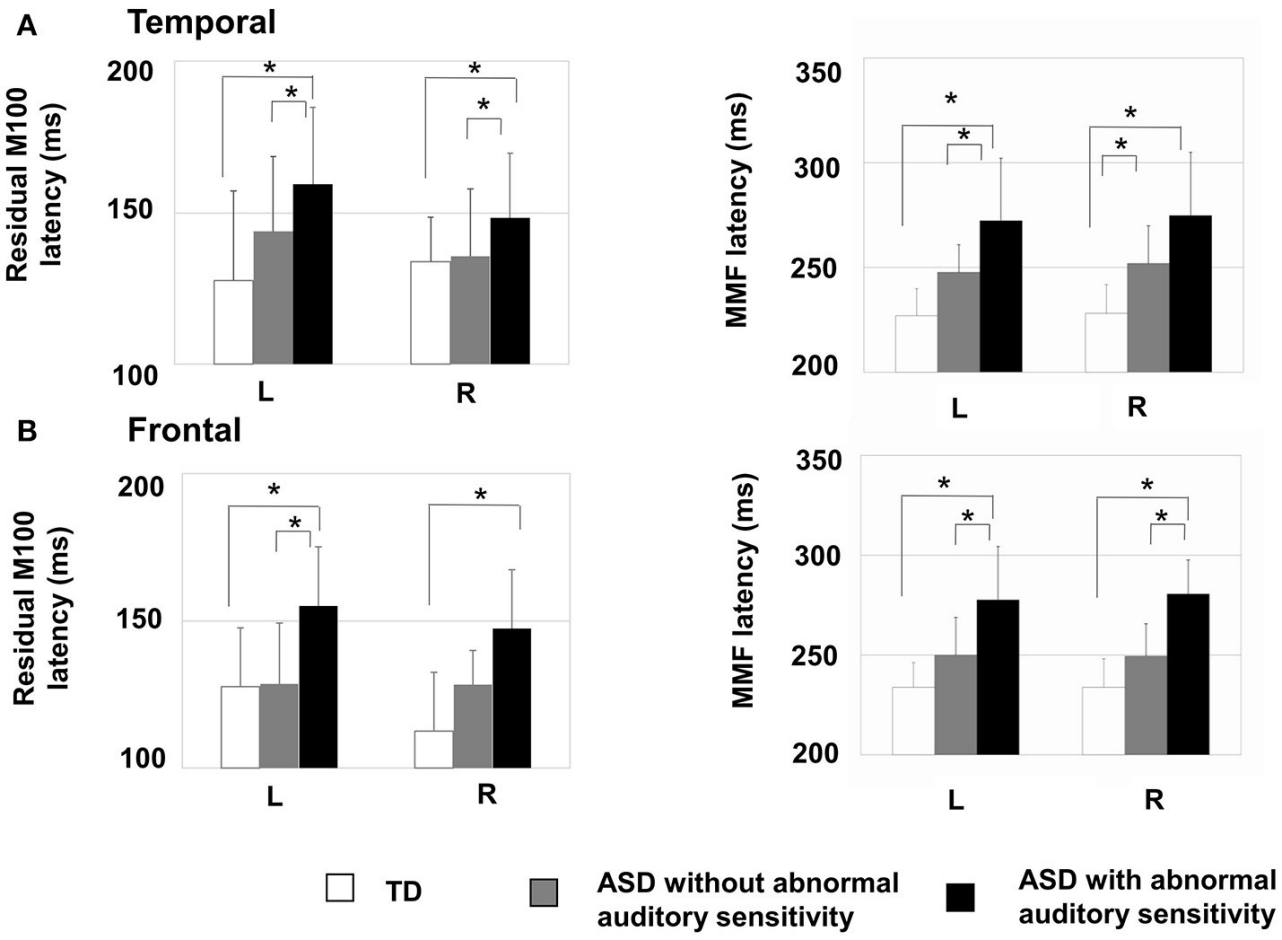
Mean residual M100 latencies and MMF latencies are presented for each hemisphere by group. Error bars represent 1 standard deviation of the mean. (**A**-Left) Residual M100 latencies in the temporal area of the autism spectrum disorder (ASD) with abnormal auditory sensitivity group were significantly longer than those in the other two groups (*p* < 0.05). (**A**-Right) MMF latencies in the temporal area of ASD with abnormal auditory sensitivity were longer than those in the other two groups (*p* < 0.05). However, comparison between ASD with and without abnormal auditory sensitivity were not statistically significant (*p* = 0.078). (**B**-Left) Individuals with ASD with abnormal auditory sensitivity exhibited significantly longer residual M100 in the frontal area compared with the frontal MMFs in the other two groups. However, comparison between ASD with and without abnormal auditory sensitivity did not reveal a statistically significant difference (*p* = 0.074). (**B**-Right) MMF latencies in the frontal area of ASD with abnormal auditory sensitivity were longer than those in the other two groups (*p* < 0.05). Asterisk indicates statistical significance (set at *p* < 0.05).

**Figure 3:**
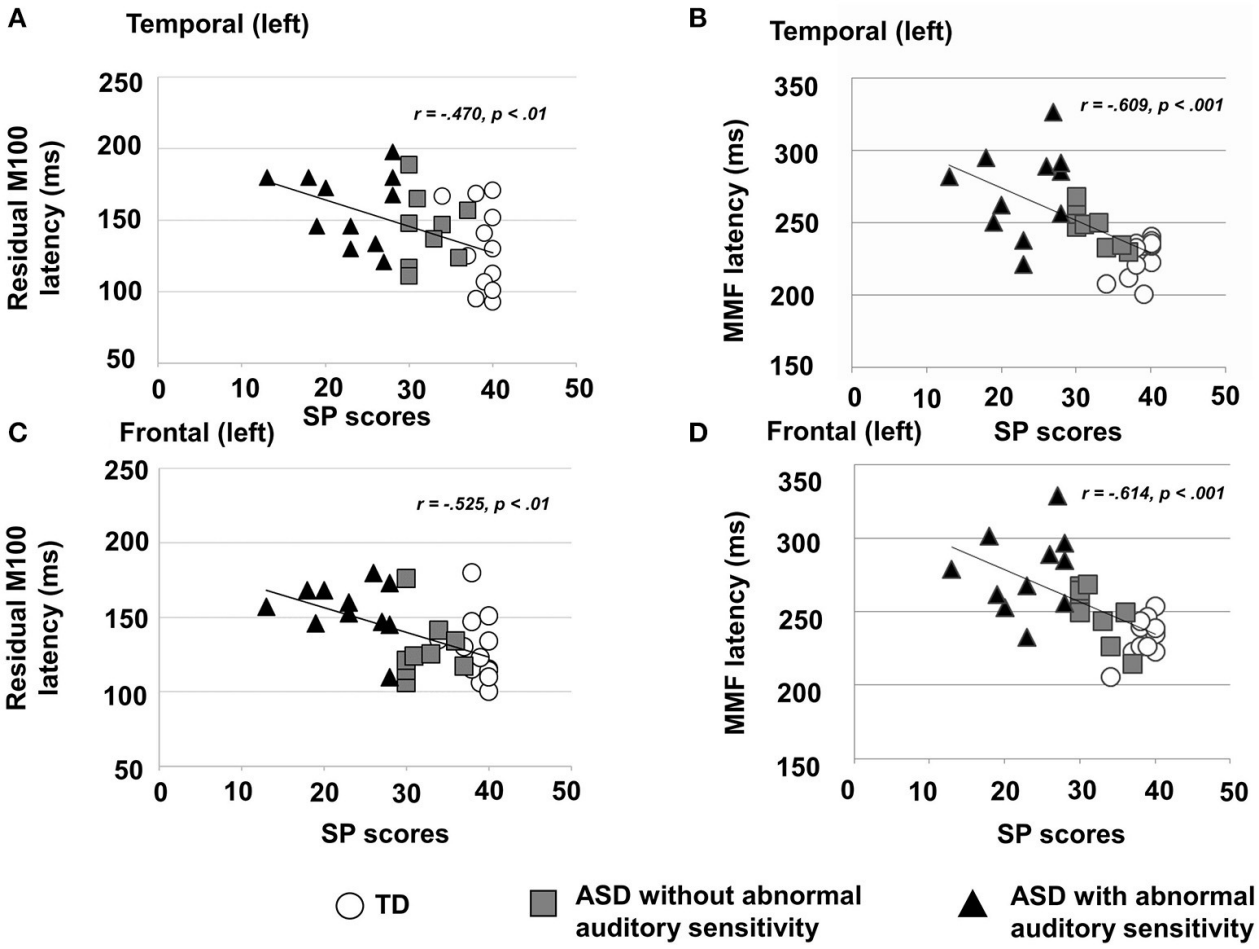
**(A)** Scatter plot of residual M100 latencies and Sensory Profile (SP) auditory item scores in the left temporal area, depicting a significant negative correlation (*p* < 0.01). **(B)** Scatter plot of right MMF latencies and SP auditory item scores in the right temporal area, depicting a significant negative correlation (*p* < 0.001). **(C)** Scatter plot of residual M100 latencies and SP auditory item scores in the left frontal area, depicting a significantly negative correlation (*p* < 0.01). **(D)** Scatter plot of MMF latencies and SP auditory item scores in the right frontal area, depicting a significant negative correlation (*p* < 0.001).

There was a significant main effect of group in the left temporal area [*F*_(2, 30)_ = 4.19, *p* = 0.025, η^2^ = 0.22; Figure 2A-left], which was not seen on the contralateral side [*F*_(2, 30)_ = 1.88, *p* = 0.170, η^2^ = 0.13]. Moreover, there was a significant main effect of group on the latency of residual M100 components in the left frontal area [*F*_(2, 30)_ = 6.31, *p* < 0.001, η^2^ = 0.50; Figure 2B-left] and right frontal area [*F*_(2, 30)_ = 10.44, *p* < 0.001, η^2^ = 0.48]. The ASD group with abnormal auditory sensitivity had longer residual M100 latencies compared with the other two groups (all *ps* < 0.05). However, comparison between ASD with and without abnormal auditory sensitivity did not reveal a statistically significant difference (*p* = 0.074) in the right frontal area. In the temporal area, there was a negative correlation between residual M100 latencies and SP scores in the left temporal area [*r* = −0.470, *p* < 0.01; Figure 3A]. In the right temporal area, there was a negative correlation between residual M100 latencies and SP scores, but this was not statistically significant [*r* = −0.232, *p* = 0.193]. In the frontal area, there was a significant negative correlation between residual M100 latencies and SP scores [left hemisphere: *r* = −0.525, *p* < 0.01; Figure 3C, right hemisphere: *r* = −0.539, *p* < 0.01].

Additionally, there was a significant positive correlation between residual M100 latencies and attention scores in the right frontal area [*r* = 0.466, *p* < 0.05]. There was also a positive correlation between attention scores and residual M100 latencies in left frontal areas, but this was not statistically significant [*r* = 0.427, *p* = 0.068]. There were no significant correlations between residual M100 latencies and any other behavioral scores.

### Residual M100 activated intensities

There was no significant main effect of group on residual M100 activated intensities in the left frontal area [*F*_(2, 30)_ = 0.52, *p* = 0.599] or right frontal area [*F*_(2, 30)_ = 0.65, *p* = 0.530]. There was also no significant main effect of group in the left temporal area [*F*_(2, 30)_ = 0.23, *p* = 0.793] or right temporal area [*F*_(2, 30)_ = 0.12, *p* = 0.884]. In the left temporal area, there was a significant negative correlation between residual M100 activated intensities and attention scores [*r* = −0.527, *p* < 0.05]. There were no significant correlations between residual M100 activated intensities and any other behavioral scores.

### MMF latencies

There was a significant main effect of group in the left temporal area [*F*_(2, 30)_ = 14.80, *p* < 0.001, η^2^ = 0.50] and right temporal area [*F*_(2, 30)_ = 13.95, *p* < 0.001, η^2^ = 0.48]. Furthermore, there was a significant main effect of group in the left frontal area [*F*_(2, 30)_ = 14.50, *p* < 0.001, η^2^ = 0.49] and right frontal area [*F*_(2, 30)_ = 26.15, *p* < 0.001, η^2^ = 0.64]. In both temporal hemispheres, the ASD group with abnormal auditory sensitivity had longer MMF latencies compared with the other two groups [*p*_*s*_ < 0.001; Figure 2A-right]. However, comparison between ASD with and without abnormal auditory sensitivity were not statistically significant in right temporal area (*p* = 0.078). In addition, individuals with ASD with abnormal auditory sensitivity had longer MMF latencies in bilateral frontal areas compared with the other two groups [*p*_*s*_ < 0.001; Figure 2B-right]. In the temporal area of both hemispheres, there was a significant negative correlation between MMF latencies and SP scores [*r* = −0.609, *p* < 0.001; Figure 3B, *r* = −0.597, *p* < 0.001]. In the frontal area of both hemispheres, there was a significant negative correlation between MMF latencies and SP scores [*r* = −0.614, *p* < 0.001; Figure 3D, *r* = −0.706, *p* < 0.001]. There was also a positive correlation between attention scores and MMF latencies in both frontal and temporal areas, however, this was not statistically significant (*p* < 0.10).

### MMF activated intensities

**Figure 4:**
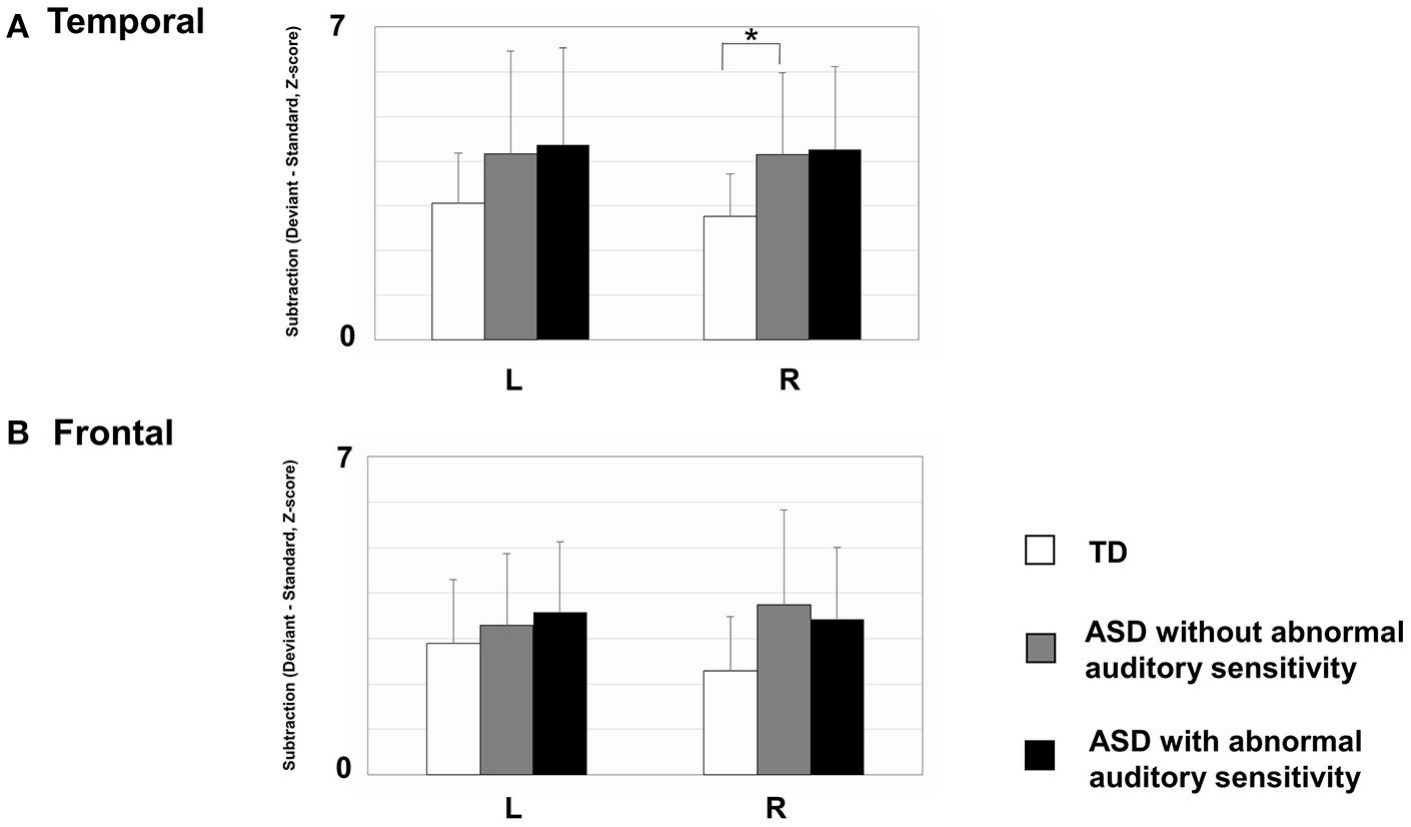
Mean mismatch field (MMF) activation intensities for each hemisphere by group. Error bars represent 1 standard deviation of the mean. **(A)** In right temporal area, those with autism spectrum disorder (ASD) showed increased activated intensities compared with typically developing (TD) children (ASD without; *p* < 0.05, ASD with; *p* = 0.062). The ASD group also exhibited increased activation intensities in the left temporal area, compared with TD participants. However, there were no significant differences between groups. **(B)** There were no differences between groups in the frontal areas of either hemisphere. Asterisk indicates statistical significance (set at *p* < 0.05).

As shown in Figure 4, there was a significant main effect of group in the right temporal area [*F*_(2, 30)_ = 3.53, *p* = 0.042, η^2^ = 0.19]. The ASD groups had increased activation intensities compared with the TD group (ASD without auditory sensitivity; *p* < 0.05, ASD with auditory sensitivity; *p* = 0.062). In addition, the ASD groups had increased activation intensities in the left temporal area compared with the TD group. However, there was no significant main effect of group [*F*_(2, 30)_ = 1.15, *p* = 0.330]. Furthermore, there was no significant main effect of group in the left frontal area [*F*_(2, 30)_ = 1.60, *p* = 0.220] or right frontal area [*F*_(2, 30)_ = 2.87, *p* = 0.073]. There were no significant correlations between activated intensities in the temporal and frontal areas of either hemisphere and any other behavioral scores.

## Discussion

The present study clearly demonstrates that abnormalities in residual M100 and MMFs (i.e., delayed latencies in the temporal and frontal areas), which reflect neural discrimination, attentional and inhibitory processing, are correlated with the severity of abnormal auditory sensitivity in children with ASD. In addition, increased MMF activated intensities were only evident in the right temporal area of both groups of children with ASD (with and without abnormal auditory sensitivity). This is the first report of an MEG study demonstrating altered MMFs to an auditory oddball paradigm located in temporal and frontal areas in patients with ASD who have abnormal auditory sensitivity.

MMFs are common variables in research investigating neurophysiological indices of auditory perception, detecting auditory change, formation of sensory memory representations, and attentional processing (Naatanen et al., 1993; Naatanen and Alho, 1995). Recently, two generators of MMNs/MMFs have been reported (Rinne et al., 2000; Lepisto et al., 2005), including a temporal generator located in the superior temporal gyrus that detects auditory changes, and a frontal generator located in the frontal gyrus that contributes to involuntary attention switching and inhibitory control processing (Cai et al., 2014). In healthy adults, shortened peak latencies to deviant stimuli have been reported, and we have also observed that TD participants and patients with ASD without abnormal auditory sensitivity have shortened peak latencies to deviant stimuli, which is consistent with findings from previous studies (Ferri et al., 2003; Kuhl et al., 2005; Lepisto et al., 2005).

Nonetheless, in a previous MMF study of ASD, Roberts et al. (2011) observed delayed MMF latencies in children with autism and language impairment. Furthermore, studies have used diffusion tensor imaging (DTI) to examine myelination, reporting atypical developmental trajectories for acoustic radiation in ASD (Roberts et al., 2013) and patients with a 16p11.2 deletion who have an increased risk of ASD (Berman et al., 2016). In addition, disruption of temporal tract connectivity has been observed only in individuals with ASD, but not in individuals without ASD but with a sensory processing disorder. Therefore, abnormal MMF responses in the temporal generator could be strongly associated with characteristic traits of ASD, and differs from the other kinds of sensory abnormality (Chang et al., 2014).

Moreover, a resting-state functional MRI (fMRI) study showed that patients with ASD had reduced connectivity between the posterior-superior temporal gyrus and many other regions, including the prefrontal cortex, striatum, amygdala, and orbitofrontal cortex (Abrams et al., 2013). Voluntary attention switching occurs via a frontoinsular-cingular attentional network that includes the anterior insula, inferior frontal gyrus, and medial frontal cortices, and this network promotes top-down modulation (Salmi et al., 2009). Frontal areas also play a crucial role in top-down inhibitory control processes (Hartmann et al., 2016) and front-temporal areas contribute to emotional regulation processes (Urbain et al., 2016). Therefore, our results showing delayed frontal MMF components suggest a dysfunction of top-down control networks and attentional impairments. Besides, children with ASD often show excessive behaviors, i.e., crying, holding hands over their ears to protect from sound, running away from sounds. Abnormalities of emotional regulation in front-temporal areas could reflect both increased and decreased behavioral reactivity to sensory stimuli in ASD children with abnormal sensitivity.

Based on these findings, the evidence suggests that abnormal auditory sensitivity may be associated with delayed myelination processes and atypical connectivity, not only in bottom-up processing, such as the primary auditory area as we reported previously (Matsuzaki et al., 2012, 2014), but also in temporal areas related with sensory processing and frontal top-down attentional and/or inhibitory control networks.

Concerning MMN amplitude, findings in several psychiatric conditions have been inconsistent, with both decreased and increased MMN amplitudes reported. For example, patients with schizophrenia have diminished frontal MMNs, which correlates with their negative symptoms, as well as temporal MMN deficits, which are associated with positive symptoms, such as auditory hallucinations (Naatanen and Kahkonen, 2009). In addition, MMN amplitudes in patients with ADHD, schizophrenia (Kemner et al., 1995), and ASD (Sawada et al., 2010) are reduced. In contrast, increased MMN amplitudes have been observed in psychiatric conditions with hyper-excitation, such as epilepsy and major depression (Rosburg et al., 2005). Activated intensities reflect the intensity of neural activation, which is a similar index to amplitudes (Naatanen et al., 1993; Strauss et al., 2015). In the present study, children with ASD had increased MMF-activated intensities in bilateral temporal areas, and there were no relationships between these activations and severity of sensory abnormalities. In contrast, decreased activated intensities for residual M100 were found in children who showed inattentive characteristics. However, the severity of sensory abnormalities and inattention were not statistically related. From these findings, we speculate that the decreased residual M100 activated-intensity might be related to characteristics of inattention, such as difficulties paying attention. Increased MMF activated-intensity might reflect autistic traits, possibly resulting from abnormal cortical excitation and dysregulation of top-down attentional switching and inhibitory control processing.

Several pharmacological studies have shown that memory-based comparison processes underlying MMN/MMF are dependent on the activity of N methyl d-aspartate (NMDA) receptors (Ehrlichmann et al., 2009). In animal models where NMDA-receptor antagonists are infused into the auditory cortex, MMN was reduced dose-dependently (Javitt et al., 1996). Moreover, dose-dependent reductions of MMN amplitude in humans has been induced by ketamine, which is also NMDA receptor antagonist (Todd et al., 2013). Schmidts (Schimidt et al., 2013) indicated that ketamine selectively reduces the normal increase in synaptic plasticity in forward connections between the primary auditory cortex and superior temporal gyrus in response to deviant tones. Therefore, NMDA receptor-related neurotransmission is critical for the generation of MMN. Recently, decreased NMDA glutamate receptor function has been reported in autistic-like social behavior in Shank2-mutant mice (Won et al., 2012). Therefore, we speculate that NMDA receptor dysfunction may contribute to abnormal sensory sensitivity in individuals with ASD.

Several studies have reported abnormal leftward lateralization of language regions in children and adolescents with ASD (Nielsen et al., 2014), as well as in adult individuals with ASD who had language delay in childhood (Floris et al., 2016). Flagg et al. (2005) also reported the presence of structural abnormalities in the left hemisphere of participants with ASD. However, we did not find any abnormal leftward or rightward lateralization in children with ASD with/without abnormal auditory sensitivity, which suggests that sensory abnormalities and language impairments in individuals with ASD may be different traits with distinct mechanisms.

There are several limitations to the present study. First, we examined a small number of stimuli, although a large number of stimuli would allow for more accurate MMF identification. However, this condition took almost 15 min including the inter-stimulus interval. Because children with ASD have difficulties keeping still for long periods, if the measurements took more time, more motion artifacts would be observed. Indeed, we were obliged to exclude some stimuli because of motion artifacts in some participants. Therefore, a balance must be struck between collecting data for a large number of stimuli and gaining usable data, without placing too much of a burden on the children. In a future study, the amount of trials presented should be increased by for example, shortening the inter-stimulus interval, and by introducing the experiment in several short blocks separated by breaks during which the children could relax and move around as they please. Second, we only performed the oddball paradigm as the auditory stimulus condition. Although difficulties in auditory language discrimination have been reported in patients with ASD (Naatanen and Alho, 1995), other tasks, such as word listening or word reading should be investigated to reveal more detailed information regarding the association between language impairments and abnormal auditory sensitivity. In addition, other tasks that include visual, multisensory, and emotional stimuli may help to clarify associations between abnormal sensitivity and neural connectivity to other related areas. Third, we only focused on auditory sensitivity, and did not assess the potential relationships between abnormal auditory sensitivity and abnormal sensitivity in other domains, delayed language acquisition, executive function, or language abilities. Such studies may elucidate subgroups of participants with ASD who have other sensory abnormalities. Finally, future studies should include more participants, including other age ranges, to elucidate the most appropriate objective physiological measures associated with sensory abnormalities in ASD. Understanding the neurological basis of these abnormalities and developing clinical tools to objectively measure them may result in effective treatments for individuals with ASD.

In summary, the present study revealed delayed MMF latencies only in ASD patients with abnormal auditory sensitivity, and these delays were correlated with the severity of abnormal auditory sensitivity. We propose that neural circuitry abnormalities in the primary auditory cortex, temporal area, and frontal attentional/inhibitory control networks are associated with abnormal auditory sensitivity in ASD. Our findings suggest that the physiological mechanisms underlying abnormal auditory sensitivity may include abnormalities in several stages of auditory processing and involve multiple neural mechanisms, such as delayed myelination processes, abnormal connectivity, as well as sensory gating system dysfunction or imbalances of inhibitory/excitatory interneurons.

## Author contributions

JM and KK designed the study. JM, KK, MH, IM, and MkT drafted the manuscript and prepared the figures. JM, KK, HS, and MH designed the experiments and performed the experiments. JM, TY, FN, RH, MyT, and KT analyzed the data. JM, TY, FN, and RH conducted the autism and cognitive assessments. The diagnostic criteria for autism were determined by MyT, KT, IM, and MkT. All authors reviewed and approved the manuscript.

### Conflict of interest statement

The authors declare that the research was conducted in the absence of any commercial or financial relationships that could be construed as a potential conflict of interest.
